# The accuracy of contrast-enhanced computed tomography scans to detect postpartum haemorrhage: an observational study

**DOI:** 10.1186/s12884-021-04306-2

**Published:** 2022-01-25

**Authors:** Yumi Mitsuyama, Yusuke Katayama, Kazuya Oi, Junya Shimazaki, Kazuya Mimura, Masayuki Endo, Takeshi Shimazu

**Affiliations:** 1 Department of Traumatology and Acute Critical Medicine, Osaka University Graduate School of Medicine, 2-15 Yamadaoka, Suita City, Osaka, 565-0871 Japan; 2 Present address: Division of Trauma and Surgical Critical Care, Osaka General Medical Centre, 3-1-56 Bandai-Higashi, Sumiyoshi-ku, Osaka, 558-8558 Japan; 3 Osaka Police Hospital, 10-31 Kitayamacho, Tennouji-ku, Osaka, 543-0035 Japan; 4 Department of Obstetrics and Gynaecology, Osaka University Graduate School of Medicine, 2-15 Yamadaoka, Suita City, Osaka, 565-0871 Japan; 5 Department of Children and Women’s Health, Graduate School of Medicine, Division of Health Science, Osaka University, Osaka, Japan

**Keywords:** Postpartum haemorrhage, Computed tomography, Extravasation, Angiography

## Abstract

**Introduction:**

Postpartum haemorrhage is a major cause of maternal mortality. Although contrast-enhanced computed tomography (CE-CT) is useful to reveal arterial bleeding, its accuracy in postpartum haemorrhage is unclear. The aim of this study was to evaluate the accuracy of CE-CT scanning in detecting postpartum haemorrhage.

**Methods:**

This was a retrospective observational study. We included patients with postpartum haemorrhage treated by emergency physicians in collaboration with obstetricians. We calculated the sensitivity, specificity, and positive and negative predictive values for CE-CT scanning to detect arterial bleeding.

**Results:**

CE-CT scanning was performed in 52 patients, and 31 patients had extravasation. The sensitivity of CE-CT scanning to detect arterial extravasation was 100% (15/15), specificity was 28.6% (2/7), positive predictive value was 75% (15/20), and negative predictive value was 100% (2/2).

**Conclusion:**

We showed the sensitivity of CE-CT scanning to detect arterial extravasation in patients with postpartum haemorrhage to be 100%.

## Introduction

Postpartum haemorrhage is a serious obstetric complication and a major cause of maternal mortality that accounts for an estimated 127,000 deaths annually [1]. The main source of bleeding is arterial, but the involvement of venous bleeding in postpartum haemorrhage is not clear [2]. As the haemorrhage progresses, coagulopathy can accelerate bleeding and make it difficult to identify the source [3]. It is very important to identify the source of bleeding to select the appropriate haemostatic treatment. Ultrasonography is appropriate for the initial imaging assessment of postpartum haemorrhage because of its minimally invasiveness and simplicity [4]. However, it is difficult to identify active bleeding and the site of haemorrhage with ultrasonography.

In the area of trauma, due to its minimal invasiveness and ease of use, contrast-enhanced computed tomography (CE-CT) is used to identify the source of bleeding [5]. Angiography is recommended in the guidelines, especially for arterial bleeding associated with pelvic fractures with stable circulation [6]. The accuracy of CE-CT and angiography has been reported to be high and CE-CT has been shown to play an important role in the detection of pelvic organ haemorrhage in the trauma setting [7]. Kawamura et al. reported that CE-CT may be useful in identifying the sites of postpartum haemorrhage [8]. However, there is little evidence on the use of CE-CT and angiography to identify the source of bleeding in postpartum haemorrhage.

The purpose of this study was to evaluate the accuracy of CE-CT in identifying the site of active bleeding in patients with postpartum haemorrhage.

## Methods

### Study design and setting

This was a retrospective observational study with a study period of 104 months from 1 April 2011 to 31 December 2019. The Trauma and Acute Critical Care Center of Osaka University Hospital, which is located in the northern part of Osaka Prefecture, Japan, annually treats about 1000 patients with conditions such as shock, cardiopulmonary arrest, severe trauma, and sepsis. The Center also treats critically ill patients with postpartum haemorrhage in cooperation with obstetricians and gynaecologists. This study included the patients who suffered haemorrhage in the postpartum period and were treated at this institution. On the basis of the diagnosis at hospital admission, we defined the patients with bleeding during the postpartum period as patients with postpartum haemorrhage. Patients experiencing bleeding due to miscarriage were excluded from the study, as were patients with eclampsia and HELLP (haemolysis, elevated liver enzymes, and low platelets) syndrome without postpartum haemorrhage. We also excluded patients who did not undergo CT scanning, were not admitted to the ICU, and were not initially treated by emergency physicians. This manuscript was written based on the STROBE statement to assess the reporting of cohort and cross-sectional studies [9]. This study was approved by the institutional review board of Osaka University (approval no. 19509). Consent from individual patients was obtained comprehensively on admission.

### Obstetric & Gynecologic Cooperative System in Osaka prefecture

In Japan, there are three levels of perinatal care depending on the risk of pregnancy and delivery [10]. Low-risk pregnancies and deliveries are managed in small hospitals and clinics, whereas pregnancies and deliveries with moderate risk, such as emergency or abnormal deliveries and caesarean sections, are managed in regional perinatal medical centres (292 facilities in Japan) that can provide emergency deliveries, caesarean section, and intensive care management for the mothers and new-borns. High-risk pregnancies and deliveries and infants requiring neonatal intensive care are managed at general perinatal medical centres (104 facilities in Japan) that are equipped with maternal-foetal intensive care units and neonatal intensive care units and can provide intensive care for the mothers, foetuses, and new-borns. In Japan, there is a system for referral and transport to higher-level medical facilities according to changes in the risk of pregnancy and delivery throughout the course of the pregnancy. In addition, the Obstetric & Gynecologic Cooperative System (OGCS) for pregnant women and the Neonatal Mutual Cooperative System for new-borns have been established in Osaka prefecture [11]. When emergency maternal events due to obstetric diseases such as massive bleeding and eclampsia, pulmonary embolism or complications related to obstetric anaesthesia occur, the OGCS allows obstetricians and gynaecologists to directly contact the obstetricians and gynaecologists at the higher-care facility for smooth transport. If serious complications such as placental abruption or severe postpartum haemorrhage occur in small hospitals and clinics, these patients can be transported to higher-level medical facilities through the OGCS for appropriate treatment. Osaka Prefecture has 17 regional perinatal medical centres and 6 general perinatal medical centres, and 34 facilities including 23 of these were registered in the OGCS. Because the most seriously ill pregnant women suffering a traffic accident, cerebral haemorrhage, or cardiopulmonary arrest cannot be adequately treated in normal medical facilities, they are transported to a hospital that has not only an emergency care centre but also a general or regional perinatal medical centre (7 facilities in Osaka Prefecture) for intensive care. Our hospital is designated as one of these institutions.

### Management of postpartum haemorrhage at Osaka University Hospital

All critical obstetric patients transported to the Trauma and Acute Critical Care Centre are handled by emergency physicians, obstetricians, and gynaecologists who work together to treat them. The obstetricians and gynaecologists receive the request for patient transfer through the OGCS, and then they notify emergency physicians, anaesthesiologists, and radiologists about the critical obstetric patients being transferred to our hospital. When a woman is transported to our critical care centre, emergency physicians resuscitate her with treatments such as blood transfusion and circulation management. At the same time, the obstetricians and gynaecologists search for a cause by examination of the genital tract and ultrasonography; tone, tissue, trauma and thrombin and stop postpartum haemorrhage with manual compression of the uterus and balloon compressions. If the vital signs are stabilized and the active bleeding disappears after these procedures, CT scanning is not performed. When the vital signs are not stabilized, active bleeding is observed, or ultrasonography shows bleeding, CT scanning should be performed.

Our emergency room includes an emergency care room equipped with a computed tomography (CT) table as a treatment bed. Because patients are treated on the CT table, it is possible to perform the entire process from diagnosis to treatment, including resuscitation, without moving them [12]. All examinations were performed using a single-source, 64-row helical scanner (SOMATOM Definition Flash, Siemens Healthineers®, Germany) with a slice thickness of 5 mm and a reconstruction interval of 5 mm during a single breath-hold. In the protocol for CE-CT scanning for postpartum haemorrhage, we use 95 mL of iohexol (Ioberin®) at an infusion rate of 4 mL/sec and the usual scanning time was 13–14 s. The area from the inferior margin of the sternum to the pelvic floor is examined in two phases of arterial and venous imaging. An Artis Q ceiling-mounted system (Siemens Healthineers®) was used for angiography.

### Endpoints

The primary endpoint was the presence of arterial bleeding. We defined extravasation on angiography as arterial bleeding in this study. The secondary endpoint was discharge to death.

### Statistical analysis

Continuous variables are indicated by medians (interquartile range [IQR]), and categorical variables are indicated by frequencies and percentages. The shock index (SI), serum lactate level, and blood loss within 24 h were used as indicators of the severity of the patients on hospital admission. The causes of postpartum haemorrhage were classified into four categories: Tone, Trauma, Tissue and Thrombin [13]. We classified atonic postpartum haemorrhage as Tone; uterine rupture, uterine inversion, cervical and vaginal lacerations, retained placenta and perineal lacerations as Trauma; placenta accreta and placenta previa as Tissue; and disseminated intravascular coagulation and amniotic fluid embolism as Thrombin. We calculated the sensitivity, specificity and positive and negative predictive values of CE-CT scanning to detect arterial bleeding to determine the accuracy of CE-CT scanning to identify the source of arterial bleeding in patients with postpartum haemorrhage. The presence of arterial extravasation on CE-CT scanning was evaluated by two independent radiologists. In addition, we performed subgroup analysis according to the patients’ SI. The accuracy of CE-CT scanning to detect arterial bleeding was similarly calculated by dividing the patients into two groups: those with SI of less than 1 and those with SI of 1 or more. The Mann-Whitney U test was used to test the continuous variables, and the Fisher exact test was used to test the nominal variables. All analyses were performed using JMP 15 (SAS Institute Inc., Cary, NC, USA).

## Results

**Fig. 1:**
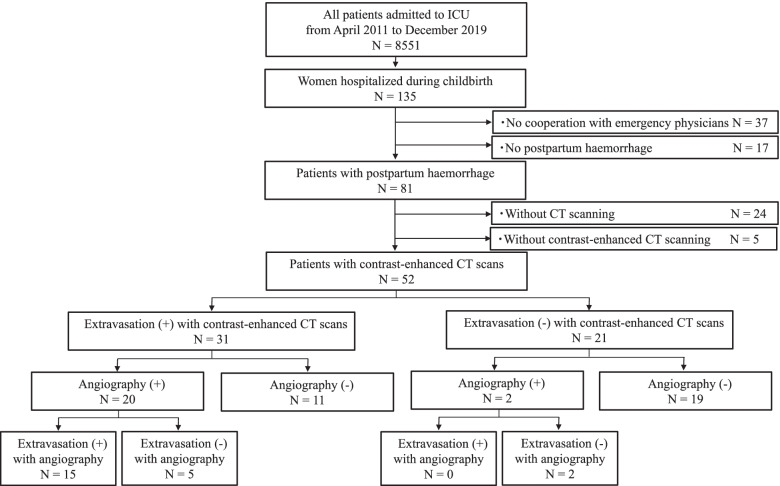
Patient flow in the study. ICU, intensive care unit; CT, computed tomography

Figure 1 shows the flow diagram of this study. Among the 81 patients with postpartum haemorrhage treated by emergency physicians in collaboration with obstetricians and gynaecologists during the study period, CE-CT scanning was performed in 52 patients. Among them, 31 patients showed extravasation on CE-CT scanning, and 21 patients showed no extravasation.

Table 1 shows the patient characteristics divided into four groups according to the presence or absence of extravasation on CE-CT scanning and angiography. The median blood loss was 3045 ml (IQR: 1557–6426 ml) in the group with extravasation on CE-CT scanning and angiography and 4011 ml (IQR: 2100–6353 ml) in the group without extravasation on CE-CT scanning and angiography.

**Table 1:** Patient characteristics

	Extravasation on CT scanning		No extravasation on CT scanning		p Value
	Angiography	No angiography	Angiography	No angiography	
	N = 20	N = 11	N = 2	N = 19	
Maternal characteristics					
Age, median (IQR)	31 (29-36)	33 (33-39)	37 (34-40)	34 (31-38)	0.24
Pre-pregnant BMI, median (IQR)	23.2 (20.4-25.2)	22.6 (20.7-22.9)	21 (21-21)	22.3 (20.1-25.9)	0.90
Assisted reproductive technologies, n (%)	3 (20.0)	3 (37.5)	1 (50.0)	5 (26.3)	0.58
Vaginal delivery, n (%)	9 (45.0)	4 (36.4)	1 (71.4)	14 (73.7)	0.16
Caesarean delivery, n (%)	5 (25.0)	4 (36.4)	0 (0)	3 (15.8)	0.44
Assisted vaginal delivery, n (%)	6 (30.0)	3 (27.2)	1 (50.0)	2 (11.1)	0.39
Post-partum haemorrhage details, n (%)					
Tone	14 (70.0)	8 (72.7)	1 (50.0)	11 (57.9)	0.77
Trauma	4 (20.0)	3 (27.3)	1 (50.0)	7 (36.8)	0.62
Tissue	1 (5.0)	0 (0)	0 (0)	1 (5.3)	0.76
Thrombin	4 (20.0)	2 (18.2)	0 (0)	1 (5.3)	0.42
Severity, median (IQR)					
Shock index	0.8 (0.7-0.9)	1 (0.9-1.1)	1.1 (0.7-1.5)	0.8 (0.7-1.0)	0.18
Lactate (mg/dl)	24 (17-32)	24 (15-43)	15 (6-24)	22 (10-33)	0.79
Blood loss (ml)	3045 (1557-6426)	4011 (2100-6353)	1132 (1109-1132)	2247 (1155-2730)	0.08
Treatment, n (%)					
Embolization	19 (95.0)	0 (0)	1 (50.0)	0 (0)	<0.01
Bilateral uterine arteries	6 (30.0)		0 (0)		
Unilateral uterine artery	4 (20.0)		1 (100.0)		
Inferior epigastric artery	3 (15.0)		0 (0)		
Internal pudendal artery	2 (10.0)		0 (0)		
Vaginal artery	2 (10.0)		0 (0)		
Perineal artery	1 (5.0)		0 (0)		
Internal iliac artery	1 (5.0)		0 (0)		
Middle rectal artery	1 (5.0)		0 (0)		
Obturator artery	1 (5.0)		0 (0)		
Surgery	2 (10.0)	5 (45.5)	0 (0)	3 (14.3)	0.11
Hysterectomy	2 (10.0)	4 (36.4)		2 (10.5)	0.22
Perineal repair	0 (0)	1 (9.1)		1 (5.3)	0.49
Postpartum balloon	9 (45.0)	8 (72.7)		7 (36.8)	0.10
Transfusion (mL), median (IQR)					
RBC suspension	1680 (560-3640)	1120 (560-2240)	420 (0-840)	840 (560-1120)	0.20
FFP	1200 (0-3480)	960 (240-2160)	240 (0-480)	480 (0-720)	0.33
PC	0 (0-400)	0 (0-400)	0 (0)	0 (0)	0.20
Disease course					
Length of ICU stay, days, median (IQR)	2 (2-3)	2 (2-3)	2 (2-2)	2 (2-2)	0.47
Length of hospital stay, days, median (IQR)	6 (4-12)	4 (3-11)	8 (5-11)	4 (3-7)	0.30
Discharge to survival, n (%)	20 (100)	11 (100)	2 (100)	19 (100)	1.00

*CT* computed tomography, *IQR* interquartile range, *BMI* body mass index, *RBC* red blood cell, *FFP* fresh frozen plasma, *PC* platelet concentrate, *ICU* intensive care unit

In the group with extravasation on CE-CT scanning and angiography, arterial embolization was performed in 19 patients (95.0%), surgery in 2 patients (10.0%), and postpartum balloon in 9 patients (45.0%). The most frequent bleeding sites identified by angiography in these patients were the uterine arteries, with bilateral uterine arteries in 6 cases and unilateral uterine arteries in 4 cases. In contrast, in the group with extravasation on CE-CT scanning and without angiography, surgery was performed in 5 patients (45.5%), and postpartum balloon in 8 patients (72.7%). In the group without extravasation on CE-CT and with angiography, embolization was performed in one patient. The site of bleeding identified by angiography was the unilateral uterine artery. In the group without extravasation on CE-CT and without angiography, surgery was performed in 3 patients (14.3%) and postpartum balloon in 7 patients (36.8%). Of the three patients, two underwent repair of perineal lacerations and rectal injuries without arterial bleeding. One patient underwent perineal hysterectomy and appendicectomy for intra-abdominal haemorrhage after cesarean section, and had no arterial bleeding. The group receiving the highest dose of RBC suspension was the group with extravasation on CE-CT and angiography, at 1680 g (IQR: 560–3640 g).

Table 2 shows the relationship between the presence of extravasation on CE-CT scanning and arterial bleeding. The sensitivity of detecting arterial extravasation on CE-CT scanning was 100% (15/15), the specificity was 28.6% (2/7), the positive predictive value was 75% (15/20), and the negative predictive value was 100% (2/2).

**Table 2:** Relationship between the presence of extravasation on contrast-enhanced CT and arterial bleeding

	Arterial bleeding		Total
	(+)	(−)	
Extravasation on CT scanning			
(+)	15	5	20
(−)	0	2	2
Total	15	7	22

*CT* computed tomography

Table 3 shows the results of the subgroup analysis of patients with SI less than 1. Of these, 18 patients underwent angiography. Twelve patients had extravasation on CE-CT scanning and 1 patient did not. The sensitivity of extravasation on CE-CT scanning for arterial bleeding was 100% (12/12), the specificity was 16.7% (1/6), the positive predictive value was 70.6% (12/17), and the negative predictive value was 100% (1/1).

**Table 3:** Cross table of extravasation on CT scanning and extravasation on angiography in patients with shock index < 1

	Arterial bleeding		Total
	(+)	(−)	
Extravasation on CT scanning			
(+)	12	5	17
(−)	0	1	1
Total	12	6	18

*CT* computed tomography

Table 4 shows the results of the subgroup analysis of patients with SI of 1 or more. Of these patients, 4 underwent angiography. The sensitivity of extravasation on CE-CT for arterial bleeding was 100% (3/3), the specificity was 100% (1/1), the positive predictive value was 100% (3/3), and the negative predictive value was 100% (1/1).

**Table 4:** Cross table of extravasation on CT scanning and arterial bleeding in patients with shock index ≥1

	Arterial bleeding		Total
	(+)	(−)	
Extravasation on CT scanning			
(+)	3	0	3
(−)	0	1	1
Total	3	1	4

*CT* computed tomography

All 52 patients with postpartum haemorrhage survived and were discharged from the hospital.

## Discussion

In the present study, we showed that in the patients with postpartum haemorrhage, the sensitivity of detecting arterial extravasation on CE-CT scanning was 100% and the negative predictive value was also 100%. It is important to identify the source of bleeding when planning a haemostatic strategy. In the case of arterial bleeding, there are options other than surgery as a haemostatic strategy, such as uterine artery embolization. Therefore, this study, which showed for the first time, to our knowledge, the accuracy of CE-CT scanning in postpartum haemorrhage, may be useful in planning strategies to treat postpartum haemorrhage.

To our best knowledge, there are no reports of the accuracy of detecting arterial extravasation on CE-CT scanning in postpartum patients. There are many reports of its utility in severe trauma patients. Salimi et al. reported that the sensitivity of CE-CT scanning for intra-abdominal organ injury was 100% for liver injury and 86.6% for splenic injury [14]. Dreizin et al. reported that the sensitivity and specificity of CE-CT scanning to search for arterial bleeding in pelvic trauma were 80 and 93%, respectively [15]. Because the presence of arterial injury can be identified by extravasation on CE-CT scanning, CE-CT scanning can rapidly identify the site of injury in parenchymal organ injury and pelvic trauma [16]. Thus, the identification of arterial extravasation with CE-CT scanning in trauma is a useful diagnostic tool due to its high sensitivity and negative predictive value [17]. Kawamura et al. reported that CE-CT may be useful in identifying the cause of postpartum haemorrhage [8]. In the present study, we collected more cases and performed statistical analysis to evaluate the sensitivity and specificity of CE-CT for arterial bleeding.

The presence of arterial bleeding is important in planning a treatment strategy because treatment options such as trans-catheter arterial embolization (TAE) are available in addition to surgical treatment. The effectiveness of uterine artery embolization for postpartum haemorrhage has also been noted in several studies [18]. It is important to establish the selection criteria for patients with postpartum haemorrhage who need TAE. H.M. Kim et al. showed that extravasation on CE-CT in the patients with postpartum haemorrhage could be an indicated condition for angiography [19]. According to the Eastern Association for Surgery of Trauma guidelines, when the cause of bleeding is intra-abdominal haemorrhage or intra-abdominal organ damage, TAE is a treatment option if the patient’s vital signs are stable [5]. Furthermore, when the cause of bleeding is retroperitoneal haemorrhage such as from pelvic fracture, TAE is the first choice for bleeding control, even if the patient’s vital signs are unstable [20]. The sensitivity of CE-CT for arterial bleeding is very high, and if the bleeding site can be accurately identified, it may be possible to select patients who need angiography.

Kuo et al. reported that in trauma with haemorrhagic shock, there were cases requiring TAE among those who did not initially show extravasation on CE-CT scanning. They reported that in patients with pelvic fractures, 19.5% (30/154) of those without extravasation on CE-CT scanning underwent TAE, and these patients were accompanied by hypotension (extravasation (+) vs. extravasation (−); 68 mmHg vs. 129 mmHg [median]) on hospital arrival [21]. In the present study, the sensitivity of detecting arterial extravasation with CE-CT scanning in postpartum haemorrhage was 100% not only in the patients with shock but also in those without shock. However, There was a patient without extravasation on CE-CT who underwent embolization. There might be false negatives on contrast-enhanced CT scans in patients with shock, in patients with temporary hemostasis due to thrombosis, or in patients with tamponade effect due to bleeding into the uterus.

Surgery is the first choice for haemostatic treatment in critical trauma patients whose vital signs are unstable. Resuscitative endovascular balloon occlusion of the aorta (REBOA) is a technique used to control subdiaphragmatic bleeding. REBOA is used as a damage control strategy for trunk trauma with haemorrhagic shock and also preoperatively and intraoperatively for surgery and transcatheter therapy. The efficacy of REBOA has been reported for postpartum haemorrhage with unstable circulation [22, 23]. In the present study, blood loss was highest in the group with extravasation on CE-CT and without angiography, (4011 ml, IQR; 2100–6353 ml), and 4 patients required hysterectomy for haemostatic treatment. Although the median SI in this group was 1.0, the condition of the patients required urgent haemostatic treatment via surgery. Hysterectomy cannot preserve fertility, but if REBOA can be used as a bridging therapy until angiography, it may be possible to preserve the uterus.

It is important to perform CE-CT scanning promptly in cases that do not respond to initial treatment or in urgent cases with unstable vital signs, and to consider the indications for angiography. CE-CT scanning is a visual imaging test that is simple and provides results in a short time. However, the level of radiation exposure has been reported to be 10–30 mSv [24]. Therefore, CE-CT scanning should not be performed in all cases of postpartum haemorrhage, but should be performed in selected cases of postpartum haemorrhage that do not respond to initial treatment. Furthermore, the long-term effects of radiation exposure on the health of young women need to be followed up.

## Limitations

There are several limitations in this study. First, this was a single-centre retrospective study and the number of cases was small, so its general validity may be low. The accuracy of CE-CT scanning should be validated by collaborating with other institutions in the future. Second, because we defined extravasation on angiography as arterial bleeding in this study, it was unknown whether the patients who showed no extravasation on CE-CT scanning and angiography actually had arterial bleeding. In cases with shock, temporary hemostasis due to thrombosis, or tamponade effect due to bleeding into the uterus, there may have been false-negative cases without extravasation on CE-CT. Moreover, of the cases with shock, CE-CT scanning was not performed in all cases, and in some cases where CT scanning was not performed, surgery was prioritized over imaging for rescue. Finally, since this study is an observational study, there may be some unknown confounding factors.

## Conclusion

The present study revealed that the sensitivity of detecting arterial extravasation on CE-CT scanning in postpartum haemorrhage was 100% and the negative predictive value was also 100%.

## Data Availability

The datasets used during the current study are available from the corresponding author on reasonable request.

## Abbreviations

CT: Computed tomography
CE-CT: Contrast-enhanced computed tomography
OGCS: Obstetric & Gynecologic Cooperative System
SI: Shock index
TAE: Trans-catheter arterial embolization
REBOA: Resuscitative endovascular balloon occlusion of the aorta
IQR: Interquartile range
